# Solitary Rectal Ulcer Syndrome Mimicking Ulcerative Colitis: A Case Report

**DOI:** 10.7759/cureus.117384

**Published:** 2026-10-03

**Authors:** Ayoub Bouziane, Btissam Essâdi, Afaf Khaled, Mohammed El Magroud, Amal Bennani, Ouiam Elmqaddam, Hajar Koulali, Abdelkrim Zazour, Zahi Ismaili, Ghizlane Kharrasse

**Affiliations:** 1 Department of Gastroenterology and Hepatology, Mohammed VI University Hospital, Oujda, MAR; 2 Research Laboratory of Pathology and Digestive Oncology, Faculty of Medicine, Pharmacy and Dental Medicine of Oujda, Mohammed First University, Oujda, MAR; 3 Department of Pathology, Mohammed VI University Hospital, Oujda, MAR

**Keywords:** dyssynergic defecation, inflammatory bowel disease, internal rectal prolapse, solitary rectal ulcer syndrome, ulcerative colitis

## Abstract

Solitary rectal ulcer syndrome (SRUS) is a benign rectal disorder associated with abnormal defecation and mucosal prolapse. Its clinical and endoscopic features may mimic inflammatory bowel disease (IBD). We report the case of a 28-year-old woman with longstanding obstructed defecation who presented with recurrent bloody mucus discharge and severe iron-deficiency anemia (hemoglobin 6.6 g/dL), which was treated with intravenous iron infusions. C-reactive protein was normal, whereas fecal calprotectin was markedly elevated. Initial colonoscopy showed erythematous and congested mucosa extending from the anal verge to 20 cm, with a large ulcer, raising suspicion of ulcerative colitis. However, the initial biopsies were inconclusive. Following clinical worsening, repeat colonoscopy revealed a 5-cm elevated rectal lesion with central ulceration. Histological examination demonstrated epithelial loss, granulation tissue, villiform crypt changes, and characteristic fibromuscular hyperplasia of the lamina propria, establishing the diagnosis of SRUS. Magnetic resonance defecography revealed an anterior rectocele and internal rectal prolapse, while anorectal manometry demonstrated dyssynergic defecation. Conservative measures and biofeedback provided incomplete improvement, with persistent rectal bleeding. Given the documented internal rectal prolapse and refractory symptoms, laparoscopic ventral rectopexy was performed, resulting in a favorable postoperative clinical course with marked symptomatic improvement and normalization of hemoglobin to 13.7 g/dL. SRUS should be considered when inflammatory-appearing rectal lesions are not supported by histological evidence of IBD, particularly in patients with obstructed defecation.

## Introduction

Lower gastrointestinal bleeding in young adults has a broad differential diagnosis, ranging from benign anorectal conditions to inflammatory, infectious, and neoplastic colorectal disease. When bleeding is accompanied by mucus discharge, tenesmus, and altered bowel habits, inflammatory bowel disease (IBD) is often considered, although rectal mucosal prolapse disorders may present with a very similar clinical and endoscopic picture.

Solitary rectal ulcer syndrome (SRUS) is a rare benign disorder, with an estimated annual incidence of approximately 1 case per 100,000 individuals. It is characterized by chronic mucosal injury, most often associated with abnormal defecatory behavior and rectal mucosal prolapse. Despite its name, SRUS does not invariably present as a solitary ulcer; its endoscopic appearance may include single or multiple ulcers, erythematous mucosa, or polypoid lesions. Common manifestations include rectal bleeding, mucus discharge, constipation, tenesmus, excessive straining, and a sensation of incomplete evacuation [1,2].

The diagnosis of SRUS can be challenging because its clinical and endoscopic features frequently overlap with those of IBD, particularly ulcerative colitis. Endoscopic findings are often nonspecific, while superficial biopsy specimens or associated inflammatory changes may lead to an erroneous diagnosis of chronic inflammatory proctitis. Therefore, an accurate diagnosis requires careful correlation between the clinical presentation, endoscopic appearance, characteristic histological findings, and the results of anorectal functional and imaging investigations [1,2].

We report a diagnostically challenging case of SRUS in a young woman with severe iron-deficiency anemia, markedly elevated fecal calprotectin, and evolving endoscopic findings that initially mimicked ulcerative colitis and rectal neoplasia.

## Case presentation

Patient information and initial presentation

A 28-year-old woman with no significant medical history presented with recurrent rectal bleeding and mucus discharge. She reported longstanding symptoms of obstructed defecation, characterized by difficult rectal evacuation, excessive straining, and a persistent sensation of incomplete evacuation. She had no extraintestinal manifestations suggestive of IBD. Her World Health Organization (WHO) performance status was 0, and abdominal examination revealed a soft, non-tender abdomen. Proctological examination showed no anal fissure, external fistulous opening, or palpable rectal mass.

Initial laboratory investigations revealed severe microcytic iron-deficiency anemia, with a hemoglobin level of 6.6 g/dL, mean corpuscular volume of 63 fL, mean corpuscular hemoglobin of 16.2 pg, and serum ferritin of 4.26 ng/mL. The patient received intravenous iron replacement therapy. There was no systemic biological inflammatory response: the white blood cell count was 6,590/mm^3^, the platelet count was 378,000/mm^3^, and C-reactive protein was within the normal range at 0.6 mg/L. Serum albumin was 40 g/L, whereas fecal calprotectin was markedly elevated at 5,106 µg/g.

Initial colonoscopy and histological assessment

Initial colonoscopy revealed continuous erythematous and congested mucosa extending from the anal verge to 20 cm, associated with a large crater-like ulcer located 15-20 cm from the anal verge. The remainder of the colonic mucosa appeared normal (Figure 1). Given the continuous distal distribution and inflammatory appearance of the lesions, ulcerative colitis was initially suspected. Histological examination of the initial rectal biopsies revealed mild chronic inflammatory changes with occasional crypt architectural distortion. However, the characteristic features of chronic active IBD, including basal plasmacytosis, diffuse crypt distortion, cryptitis, and crypt abscesses, were absent. One of the biopsy specimens was superficial and showed mucosal ulceration covered by fibrinoleukocytic exudate, which limited its diagnostic value. No granulomas, dysplasia, or malignant cells were identified. Although the mild chronic inflammation and focal architectural abnormalities could initially raise the possibility of IBD, the absence of basal plasmacytosis and active crypt injury did not support a definitive diagnosis of ulcerative colitis. Consequently, the initial histological findings were considered nonspecific, and clinicopathological reassessment was required.

**Figure 1:**
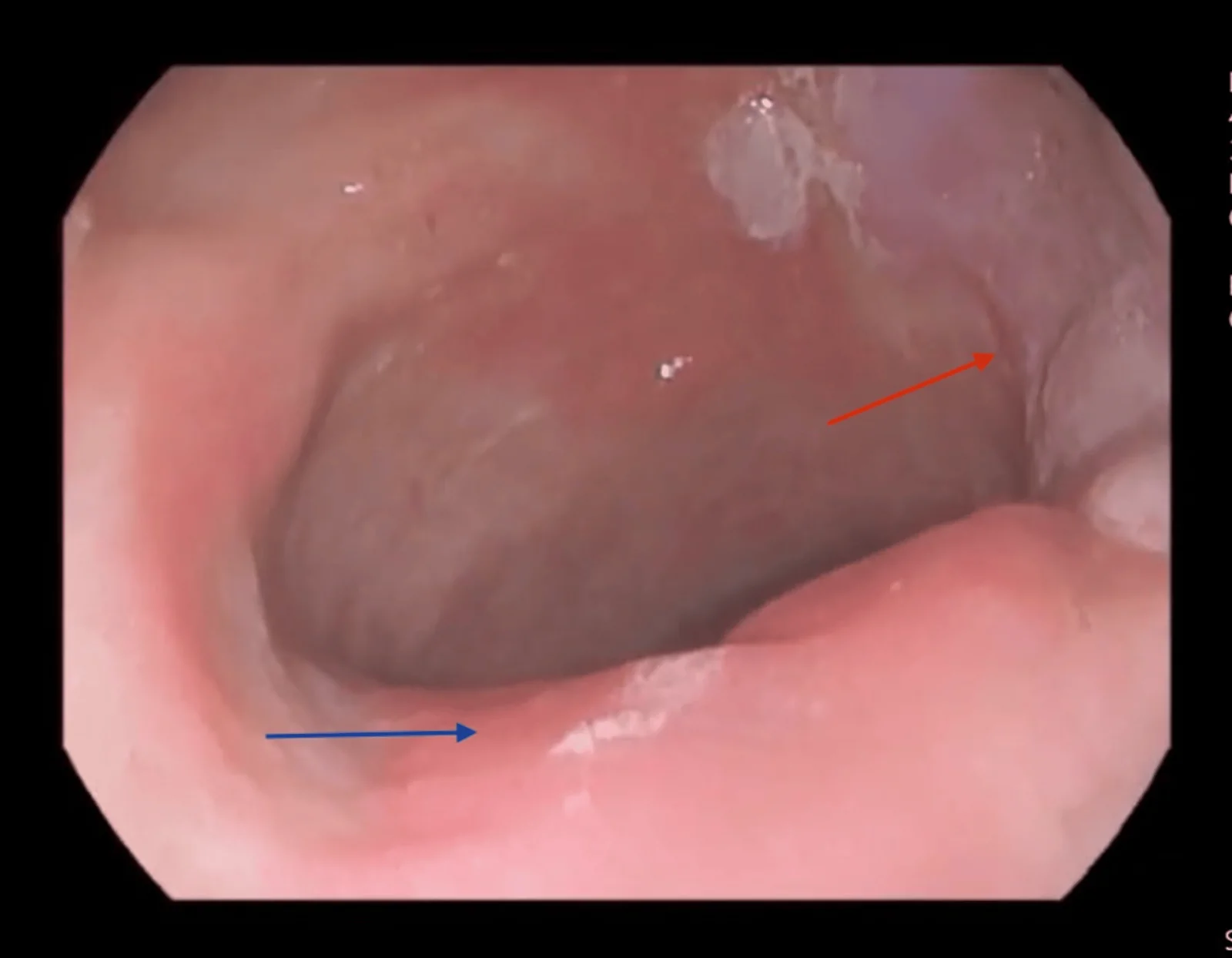
Initial colonoscopic appearance Initial colonoscopic view showing erythematous and congested rectal mucosa (blue arrow), with a partially visualized ulcer on the right side of the image (red arrow). The ulcer was located 15-20 cm from the anal verge.

Clinical course and diagnostic reassessment

The patient was lost to follow-up for eight months and subsequently returned with worsening symptoms, including up to 10 episodes of bloody mucus discharge per day. Repeat colonoscopy revealed a 5-cm elevated rectal lesion with central ulceration, located approximately 10 cm from the anal verge, while the remaining colorectal mucosa appeared normal (Figure 2). Histological examination of multiple targeted biopsies demonstrated fragments of polypoid rectal mucosa with extensive superficial epithelial loss replaced by granulation tissue (Figure 3B). The residual mucosa exhibited villiform crypt changes, focal crypt dilatation, and characteristic fibromuscular hyperplasia of the lamina propria (Figure 3A), associated with a moderate polymorphous inflammatory infiltrate. These findings were diagnostic of mucosal prolapse syndrome and, in the appropriate clinical and endoscopic context, established the diagnosis of SRUS. Additional rectal mucosal samples showed largely preserved crypt architecture with only occasional branching and mild chronic inflammation. Basal plasmacytosis, cryptitis, crypt abscesses, and granulomas were absent, providing no histological evidence of IBD. Ileal biopsies also demonstrated preserved villous architecture without granulomas, dysplasia, or other significant abnormalities.

**Figure 2:**
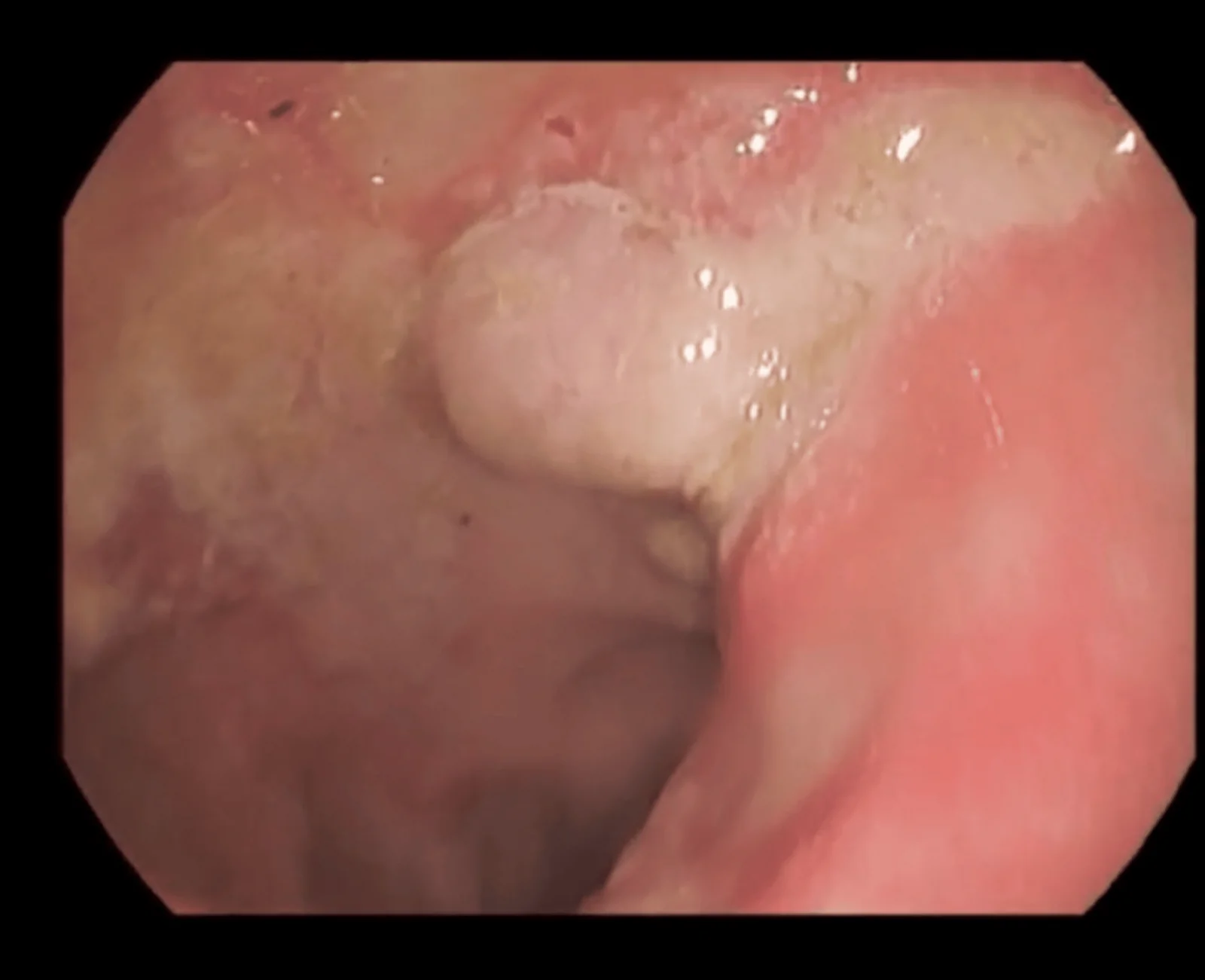
Repeat colonoscopy Repeat colonoscopy showing a 5-cm elevated rectal lesion with central ulceration, located approximately 10 cm from the anal verge.

**Figure 3:**
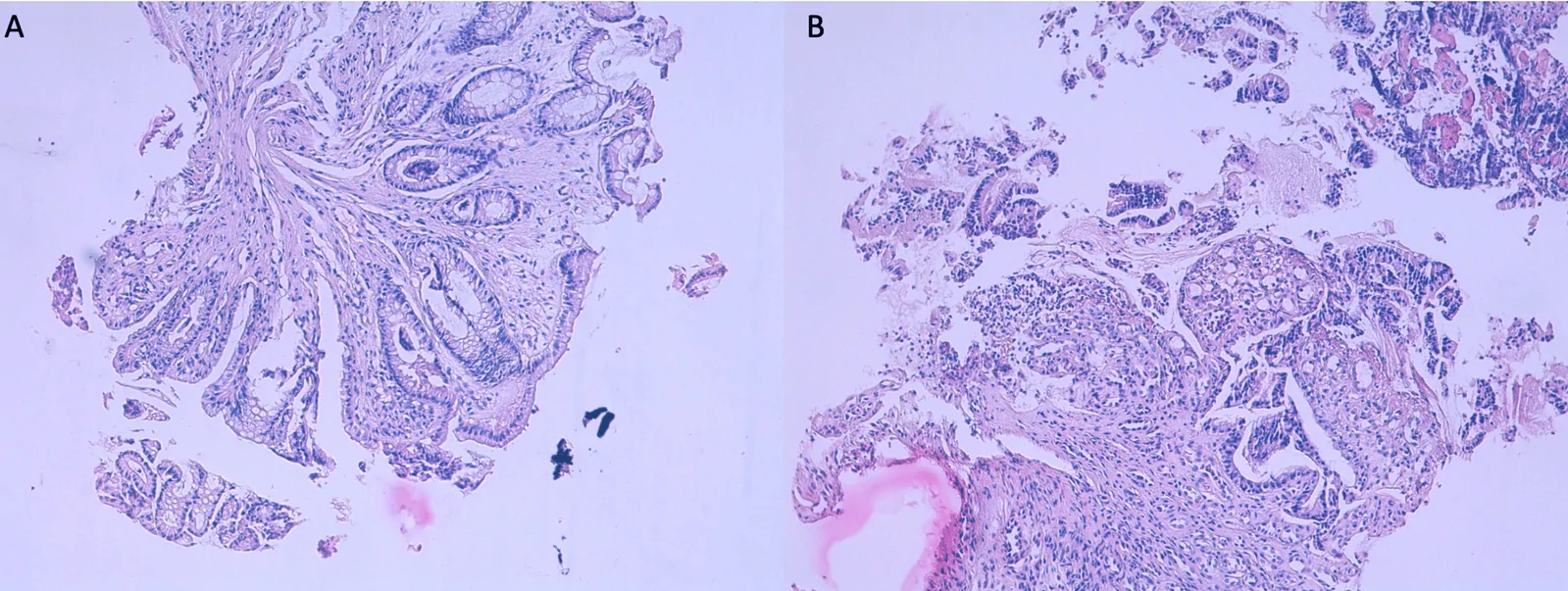
Histopathological findings from repeat colonoscopy (A) Polypoid rectal mucosa showing villiform changes, crypt dilatation, and fibromuscular hyperplasia of the lamina propria (original magnification ×100). (B) Ulcerated rectal mucosa showing reactive capillary proliferation, active fibroblasts, and a moderately dense polymorphous inflammatory infiltrate (original magnification ×200). Hematoxylin-eosin-saffron (HES) staining.

Following the histological diagnosis of SRUS, further investigations were performed to identify the underlying defecatory disorder. Magnetic resonance defecography demonstrated an anterior rectocele with incomplete terminal evacuation and internal rectal prolapse, together with asymmetric right-sided rectal wall thickening (Figure 4). Anorectal manometry subsequently showed a preserved rectoanal inhibitory reflex and normal resting sphincter pressure, but demonstrated dyssynergic defecation, reduced rectal capacity, and a shortened voluntary anal contraction. These functional and morphological abnormalities provided a coherent pathophysiological explanation for the repetitive mucosal trauma and confirmed the association between SRUS, internal rectal prolapse, and an obstructed defecation syndrome. The case was subsequently reviewed during a multidisciplinary meeting involving gastroenterologists, pathologists, radiologists, and colorectal surgeons.

**Figure 4:**
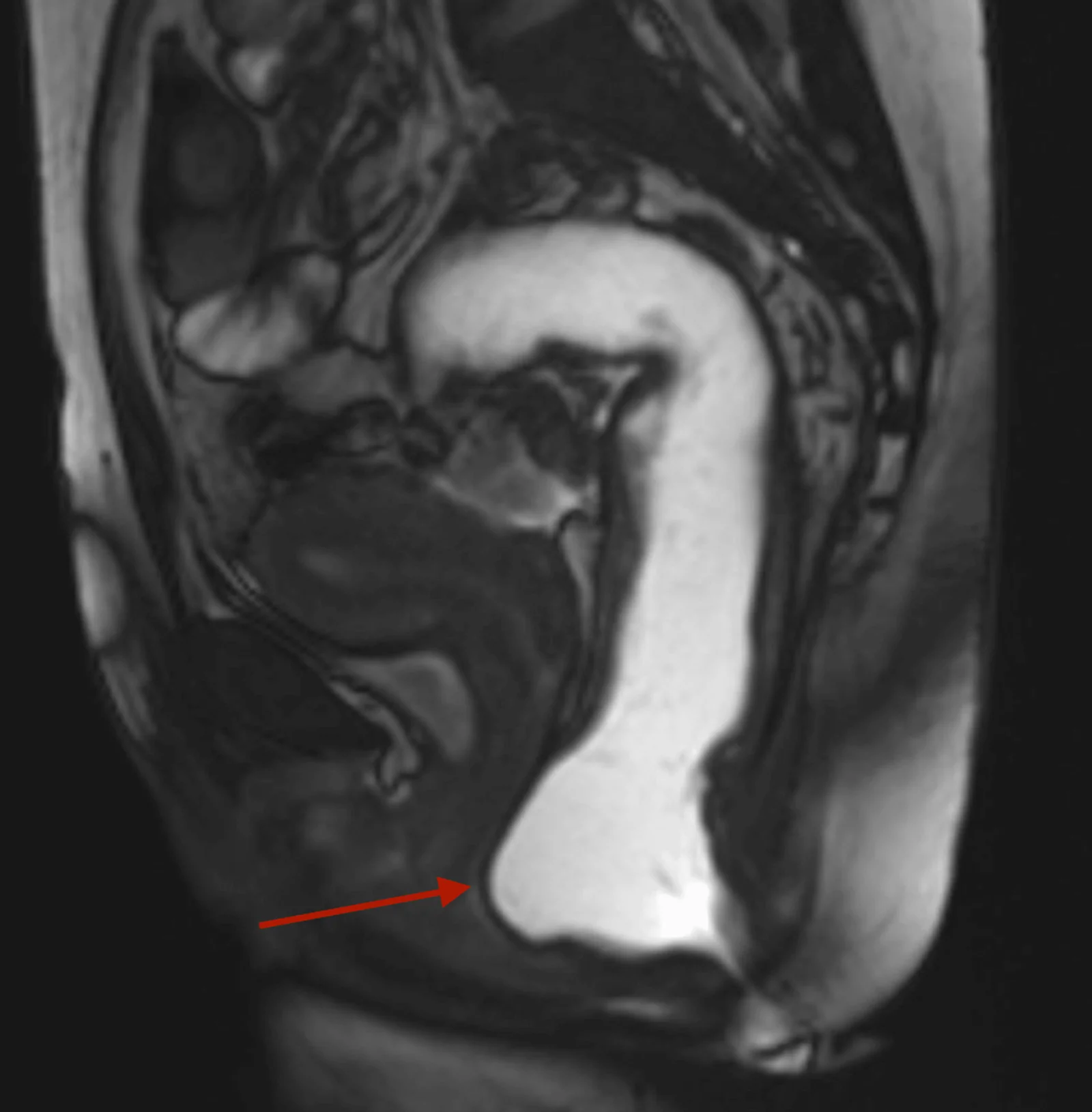
Magnetic resonance defecography Sagittal image obtained during the evacuation phase of magnetic resonance defecography, showing an anterior rectocele (red arrow).

Management and clinical response

Initial management consisted of dietary and behavioral measures aimed at improving bowel habits, reducing excessive straining, and facilitating stool evacuation, together with medical treatment to regulate intestinal transit. Because these measures failed to provide meaningful clinical improvement, the patient underwent 10 sessions of pelvic floor biofeedback therapy targeting the documented dyssynergic defecation. Biofeedback resulted in a substantial reduction in obstructive defecatory symptoms; however, intermittent rectal bleeding and mucus discharge persisted. Topical mesalamine therapy was subsequently introduced but achieved only a partial and transient response. Despite continued conservative treatment, the patient experienced recurrent rectal bleeding, occurring both during and independently of defecation, with persistent pelvic discomfort and frequent mucus discharge. The incomplete response to medical treatment and biofeedback, in the presence of documented internal rectal prolapse, prompted further endoscopic reassessment and referral for colorectal surgical evaluation. A third colonoscopy was performed because of persistent symptoms and demonstrated erythematous rectosigmoid mucosa with a superficial ulcer, together with a 1-cm polypoid lesion located 18 cm from the anal verge. Histological examination of biopsies obtained from the ulcer margins confirmed changes consistent with SRUS, whereas the polypoid lesion was identified as a hyperplastic polyp. No histological features supporting IBD, dysplasia, or malignancy were detected. Pelvic magnetic resonance imaging subsequently demonstrated asymmetric fibrous thickening of the right rectal wall, measuring up to 12 mm in thickness and extending over approximately 30 mm, at a distance of 9 cm from the anal verge. Mural stratification was preserved, without suspicious contrast enhancement or restricted diffusion, and the mesorectal fat remained intact. These radiological findings were consistent with benign fibrotic changes related to chronic mucosal prolapse and showed no evidence of rectal malignancy or active IBD. Given the persistence of clinically significant symptoms despite conservative therapy and biofeedback, together with the documented internal rectal prolapse, surgical correction was considered the most appropriate therapeutic option.

Surgical treatment and outcome

The patient underwent laparoscopic ventral rectopexy, with fixation of a non-absorbable mesh to the anterior rectal wall and sacral promontory to correct the internal rectal prolapse. The postoperative course was uncomplicated and clinically favorable, with marked improvement in obstructed defecation, pelvic discomfort, and the frequency of bloody mucus discharge. Occasional minor bleeding persisted but was considerably reduced compared with the preoperative period. Follow-up laboratory investigations showed complete correction of the iron-deficiency anemia following intravenous iron replacement and control of chronic rectal blood loss, with a hemoglobin level of 13.7 g/dL and a normal mean corpuscular volume. C-reactive protein remained normal at 0.7 mg/L, serum albumin was 50 g/L, the white blood cell count was 4,100/mm^3^, and the platelet count was 322,000/mm^3^. The favorable clinical and biological evolution supported the effectiveness of surgical correction of the underlying rectal prolapse after failure of conservative and functional treatment.

## Discussion

SRUS is a rare benign disorder characterized by chronic injury to the rectal mucosa, generally associated with abnormal defecatory behavior and mucosal prolapse. Its true prevalence is probably underestimated because of frequent diagnostic delays and misclassification as IBD or rectal neoplasia. SRUS can occur at any age but predominantly affects young and middle-aged adults; in a retrospective series of 116 patients, the mean age at diagnosis was 37.4 years [1-3]. Its pathogenesis is multifactorial and involves excessive straining, paradoxical pelvic floor contraction, digital rectal manipulation, and internal rectal prolapse. These abnormalities expose the rectal mucosa to repetitive mechanical trauma, venous congestion, ischemia, and impaired local blood flow, ultimately resulting in mucosal ulceration, inflammation, and fibrosis [1,2]. In our patient, the combination of longstanding obstructed defecation, dyssynergic defecation, an anterior rectocele, and internal rectal prolapse provided a clear pathophysiological explanation for the development and persistence of the rectal lesions.

The clinical presentation of SRUS is heterogeneous and frequently overlaps with that of IBD, particularly ulcerative colitis. Common manifestations include rectal bleeding, mucus discharge, tenesmus, constipation, excessive straining, and a persistent sensation of incomplete evacuation. Some patients report frequent small-volume evacuations that may be incorrectly interpreted as inflammatory diarrhea. In the series by Abid et al., rectal bleeding was reported in 82% of patients, constipation in 23%, and diarrhea-like symptoms in 22% [3]. Our patient presented with up to 10 episodes of bloody mucus discharge per day, initially suggesting active inflammatory proctosigmoiditis. However, the longstanding history of obstructed defecation, together with excessive straining and incomplete evacuation, favored an underlying functional defecatory disorder. This distinction is important because repeated passage of small amounts of blood and mucus does not necessarily represent true diarrhea and may instead result from tenesmus and repeated attempts at evacuation. Careful characterization of bowel habits is therefore essential when differentiating SRUS from ulcerative colitis.

The endoscopic spectrum of SRUS is broad and represents an additional source of diagnostic difficulty. Despite its name, the condition does not invariably present as a single rectal ulcer. Lesions may be solitary or multiple and may appear as erythematous mucosa, shallow or deeply ulcerated lesions, nodular thickening, or polypoid masses that resemble inflammatory or neoplastic disease. Abid et al. reported ulcerative lesions in 78% of patients and polypoid or nodular lesions in 25% [3], while Chiang et al. emphasized the considerable variability in endoscopic appearance and its potential to cause diagnostic confusion [4]. In our patient, the initial colonoscopy demonstrated continuous erythematous and congested mucosa extending from the anal verge to 20 cm, with a large crater-like ulcer, closely mimicking ulcerative proctosigmoiditis. Eight months later, the lesion had evolved into a focal 5-cm elevated mass with central ulceration surrounded by otherwise normal mucosa, raising concern for a rectal neoplasm. This changing appearance illustrates why endoscopy alone cannot reliably distinguish SRUS from IBD or malignancy. Moreover, some lesions were located more proximally than is typical for SRUS. Although SRUS lesions usually occur on the anterior rectal wall, approximately 3-10 cm from the anal verge [5], our patient had lesions extending to 15-20 cm and a subsequent polypoid lesion at 18 cm. Therefore, an atypical or relatively proximal location should not exclude SRUS when characteristic histological features are present.

Histological examination is the cornerstone of diagnosis and is essential for distinguishing SRUS from IBD. Ulcerative colitis is characterized by diffuse and continuous chronic mucosal inflammation associated with crypt architectural distortion, basal plasmacytosis, and, during active disease, cryptitis or crypt abscesses [6,7]. In contrast, the characteristic histological features of SRUS include fibromuscular obliteration or hyperplasia of the lamina propria, hypertrophy and disorganization of the muscularis mucosae, and extension of smooth muscle fibers between distorted crypts. Surface ulceration, granulation tissue, vascular congestion, reactive epithelial changes, and crypt distortion may also occur and can create histological overlap with IBD [1,2,4]. In our patient, the initial superficial biopsies showed only mild chronic inflammation and occasional crypt distortion and were therefore inconclusive. However, the deeper and more representative biopsies obtained from the elevated ulcerated lesion demonstrated polypoid mucosa with extensive epithelial loss, granulation tissue, villiform crypt changes, focal crypt dilatation, and characteristic fibromuscular hyperplasia of the lamina propria. In the absence of basal plasmacytosis, cryptitis, crypt abscesses, or granulomas, these findings established the diagnosis of SRUS within the spectrum of mucosal prolapse syndrome. This case illustrates the importance of obtaining multiple sufficiently deep biopsies from both the ulcer margins and the adjacent abnormal mucosa when SRUS is suspected, because superficial samples may contain only nonspecific inflammatory or ulcerative changes and may delay the correct diagnosis.

Defecographic imaging and anorectal physiological testing are particularly valuable for identifying the underlying mechanisms responsible for SRUS in patients with obstructed defecation. Defecography may demonstrate internal rectal intussusception, rectocele, incomplete evacuation, excessive perineal descent, or overt rectal prolapse, whereas anorectal manometry evaluates sphincter function, rectal sensation, and rectoanal coordination [1,2]. In a series of 28 patients with histologically confirmed SRUS, Abdelatty et al. reported internal rectal intussusception in 16 patients and demonstrated the usefulness of magnetic resonance defecography in identifying abnormalities associated with mucosal prolapse [8]. In our patient, magnetic resonance defecography revealed an anterior rectocele with incomplete terminal evacuation and internal rectal prolapse, while anorectal manometry demonstrated dyssynergic defecation and reduced rectal capacity. These abnormalities provided a plausible mechanical explanation for the repetitive mucosal trauma documented histologically. Pelvic magnetic resonance imaging additionally showed benign fibrous thickening of the rectal wall without restricted diffusion, suspicious enhancement, mesorectal infiltration, or other features suggestive of malignancy or IBD. Although the diagnosis was established histologically, the imaging and functional findings identified the underlying pathophysiological mechanism, guided treatment, and supported the decision to correct the internal prolapse surgically.

The differential diagnosis of SRUS includes ulcerative colitis, Crohn’s disease, infectious or ischemic proctitis, endometriosis, and benign or malignant rectal neoplasms [1,2,9]. In this case, ulcerative colitis was initially considered because of the frequent bloody mucus discharge, markedly elevated fecal calprotectin, and continuous inflammatory appearance of the distal rectal mucosa. However, repeated biopsies did not demonstrate the characteristic histological features of chronic active ulcerative colitis, while representative samples from the ulcerated lesion showed diagnostic features of mucosal prolapse syndrome. Crohn’s disease was also considered; although the absence of granulomas alone cannot exclude it, there were no supportive clinical, endoscopic, histological, or radiological features. The elevated ulcerated lesion observed during the second colonoscopy additionally raised concern for rectal malignancy. Nevertheless, repeated biopsies showed no dysplasia or malignant cells, and pelvic magnetic resonance imaging demonstrated preserved mural stratification without restricted diffusion, suspicious enhancement, or mesorectal involvement. Because SRUS may closely resemble rectal cancer endoscopically and may occasionally coexist with other colorectal disorders, adequate deep biopsy sampling and radiological correlation remain essential whenever a mass-like lesion is encountered [3,4]. In our patient, the concordance between diagnostic histology, internal rectal prolapse, and dyssynergic defecation allowed the alternative diagnoses to be reasonably excluded.

Treatment of SRUS should be individualized according to symptom severity, the presence of a defecatory disorder, and the existence of internal or external rectal prolapse. Initial management is conservative and includes patient education, adequate dietary fiber and fluid intake, regulation of bowel habits, avoidance of excessive straining and digital manipulation, and treatment of constipation [1,2,9]. Biofeedback therapy is particularly appropriate when pelvic floor dyssynergia is documented because it aims to restore coordinated rectoanal relaxation and reduce repetitive mucosal trauma. In a prospective study of 16 patients, Jarrett et al. reported symptomatic improvement in 12 patients following biofeedback, although complete endoscopic ulcer healing occurred in only five [10]. Similarly, our patient experienced substantial improvement in obstructed defecation after 10 biofeedback sessions, but rectal bleeding and mucus discharge persisted. Topical mesalamine produced only a partial response, consistent with the limited and heterogeneous evidence supporting topical therapies in SRUS [1,9]. Because clinically significant symptoms persisted despite conservative management and biofeedback, and magnetic resonance defecography had demonstrated internal rectal prolapse, laparoscopic ventral rectopexy was performed to correct the underlying anatomical abnormality. The postoperative course was favorable, with marked clinical improvement and correction of the iron-deficiency anemia. Surgical treatment should therefore be reserved for carefully selected patients with refractory symptoms and a demonstrable prolapse-related mechanism. Nevertheless, long-term outcomes remain variable, and persistent or recurrent symptoms have been reported after surgery [11], emphasizing the need for continued clinical follow-up.

This report has several limitations. As a single-patient observation, its findings cannot be generalized to all patients with SRUS, particularly because the clinical, endoscopic, and functional manifestations of this disorder are highly heterogeneous. The unusually proximal extension of some lesions and the markedly elevated fecal calprotectin represented atypical features that initially complicated the distinction from ulcerative colitis. Nevertheless, the diagnosis was established through representative histological specimens demonstrating characteristic fibromuscular changes of mucosal prolapse syndrome and was further supported by the identification of internal rectal prolapse and dyssynergic defecation. The principal strength of this case lies in the comprehensive clinicopathological, radiological, and functional assessment, which demonstrates how SRUS can mimic both IBD and a rectal neoplasm and highlights the importance of correcting the underlying defecatory disorder rather than relying solely on anti-inflammatory treatment.

## Conclusions

SRUS should be considered in patients presenting with recurrent bloody mucus discharge and inflammatory or mass-like rectal lesions, particularly when symptoms of obstructed defecation are present. Although its clinical, endoscopic, and biomarker profile may closely mimic ulcerative colitis, the diagnosis depends on adequate histological sampling demonstrating characteristic features of mucosal prolapse syndrome. Defecography and anorectal manometry are complementary investigations that identify associated anatomical and functional abnormalities, including internal rectal prolapse and dyssynergic defecation. Management should target the underlying defecatory disorder through conservative measures and biofeedback, while surgical correction may provide a favorable outcome in selected patients with persistent symptoms and documented rectal prolapse.
